# Conventional rotator cuff versus all-suture anchors—A biomechanical study focusing on the insertion angle in an unlimited cyclic model

**DOI:** 10.1371/journal.pone.0225648

**Published:** 2019-11-27

**Authors:** Dimitris Ntalos, Kay Sellenschloh, Gerd Huber, Daniel Briem, Klaus Püschel, Michael M. Morlock, Karl-Heinz Frosch, Florian Fensky, Till Orla Klatte

**Affiliations:** 1 Department of Trauma-, Hand-, and Reconstructive Surgery, University Medical Center Hamburg-Eppendorf, Hamburg, Germany; 2 Institute of Biomechanics, TUHH Hamburg University of Technology, Hamburg, Germany; 3 Asklepios Westklinikum Hamburg, Hamburg, Germany; 4 Institute of Forensic Medicine, University Medical Center Hamburg-Eppendorf, Hamburg, Germany; University of California Los Angeles, UNITED STATES

## Abstract

**Purpose:**

The purpose of this study was to compare the biomechanical properties of an all-suture anchor to a conventional anchor used commonly in rotator cuff repairs. Furthermore, the biomechanical influence of various implantation angles was evaluated in both anchor types in a human cadaveric model.

**Methods:**

30 humeri were allocated into three groups with a similar bone density. The two different anchor types were inserted at a predefined angle of 45°, 90° or 110°. Biomechanical testing included an initial preload of 20N followed by a cyclic protocol with a stepwise increasing force of 0,05N for each cycle at a rate of 1Hz until system failure. Number of cycles, maximum load to failure, stiffness, displacement and failure mode were determined.

**Results:**

27 anchors failed by pullout. There was no significant difference between the conventional and the all-suture anchor regarding mean pullout strength. No considerable discrepancy in stiffness or displacement could be perceived. Comparing the three implantation angles no significant difference could be observed for the all-suture or the conventional anchor.

**Conclusion:**

All-suture anchors show similar biomechanical properties to conventional screw shaped anchors in an unlimited cyclic model. The exact insertion angle is not a significant predictor of failure.

## 1. Introduction

Arthroscopic rotator cuff repairs using suture anchors is a widely accepted technique, yielding good clinical and functional results. Still, failure of said repairs remains a problem with the bone-anchor interface as a critical side [1–4]. Specifically, the design of the anchor is widely discussed [5,6]. Traditionally, conventional screw-shaped anchors are used for suture-fixation in the greater tuberosity. Currently, a repair technique using all-suture anchors (ASA) is receiving more attention [7–10]. All-suture anchors are entirely composed of suture material and rely on a different bone-anchor fixation mechanism. Instead of a screw-like fixation, they typically expand in the bone and are fixated underneath the cortex [7,11,12]. Preservation of native bone material through the use of smaller pilot holes is claimed to be a main advantage [13].

There are just a few studies comparing the biomechanical properties of these two anchor types in a human model, with diverging results. Mazzoca et al. and Goshka et al. showed comparable biomechanical properties whereas Nagra et al. demonstrated a decreased failure load and an increased displacement in all-suture anchors. All of these studies used testing protocols with a limited number of cycles and linear pullout forces. [7,12,14]. Therefore, they are not able to identify seating or fatigue phenomena as caused by cyclic loading, reflecting a typical rehabilitative process following rotator cuff repair.

The occurrence of incidental anchor pullout, a common mechanism of repair failure, is furthermore influenced by bone quality and the implantation technique [5,6]. Burkhart et al. repeatedly report an optimum anchor implantation angle of 45° [15–18]. In contrast Clevenger, Green as well as Strauss et al. demonstrated superior biomechanical properties at an angle of 90° or more. All published data focuses on conventional anchor systems [3,5,19]. Since these systems consist of a straight, elongated shape, a biomechanical impact of the insertion angle seems to be inevitable. All-suture anchors however have a different morphology and anchorage within the bone. Therefore, one may hypothesize that there will not be an impact of different insertion angles due to the rather round, knot-like shape.

The purpose of this study was to evaluate the biomechanical differences between all-suture and conventional anchors when subjected to different insertion angles. Additionally, in contrast to previous studies, experiments were performed using an unlimited cyclic human cadaveric shoulder model, yielding results which can be more accurately transferred to in vivo conditions.

## 2. Methods

36 human humeri (18 matched pairs) were collected from donors, between the age of 22–76 years (mean 61.4 years, standard deviation 11 years).

Approval by the Institutional Review Board was obtained specifically for this study (Ethical Review Committee Hamburg, Germany; study number: WF-27/17). The donors consent to the postmortem tissue donation was given in written form prior to death and/or by the next-of kin of the deceased person.

The specimens were sealed in plastic and stored at −20 °C.

Each was scanned using a 16 row CT-scanner (Brilliance 16 CT; Philips Healthcare, Hamburg, Germany) with a solid calibration phantom (Bone Density Calibration Phantom; QRM, Moehrendorf, Germany) to determine the volumetric bone mineral density (vBMD) in terms of calcium hydroxylapatite (mgCaHA per cm^3^) (Avizo 5.1, VSG Inc., Burlington, Massachusetts) [20,21]. Volumetric bone mineral density was determined for each humeral head within the region of interest (ROI). The ROI was defined as the volume calculated using the diameter of the humeral head at a distance of 1.5 cm distal from the tip of the greater tubercle. Osteoporotic samples with a vBMD <80 mg/cm^3^ were excluded.

Three groups of five matched pairs (n = 10 in each group) with similar mean vBMD were formed. Two different anchor types were tested on each matched pair. The Y-Knot RC^®^ (ConMed, New York, New York) an all-suture anchor with a drill size of 2.8mm and the conventional 4.50mm CrossFT^™^ Suture Anchor (ConMed, New York, New York).

**Fig 1:**
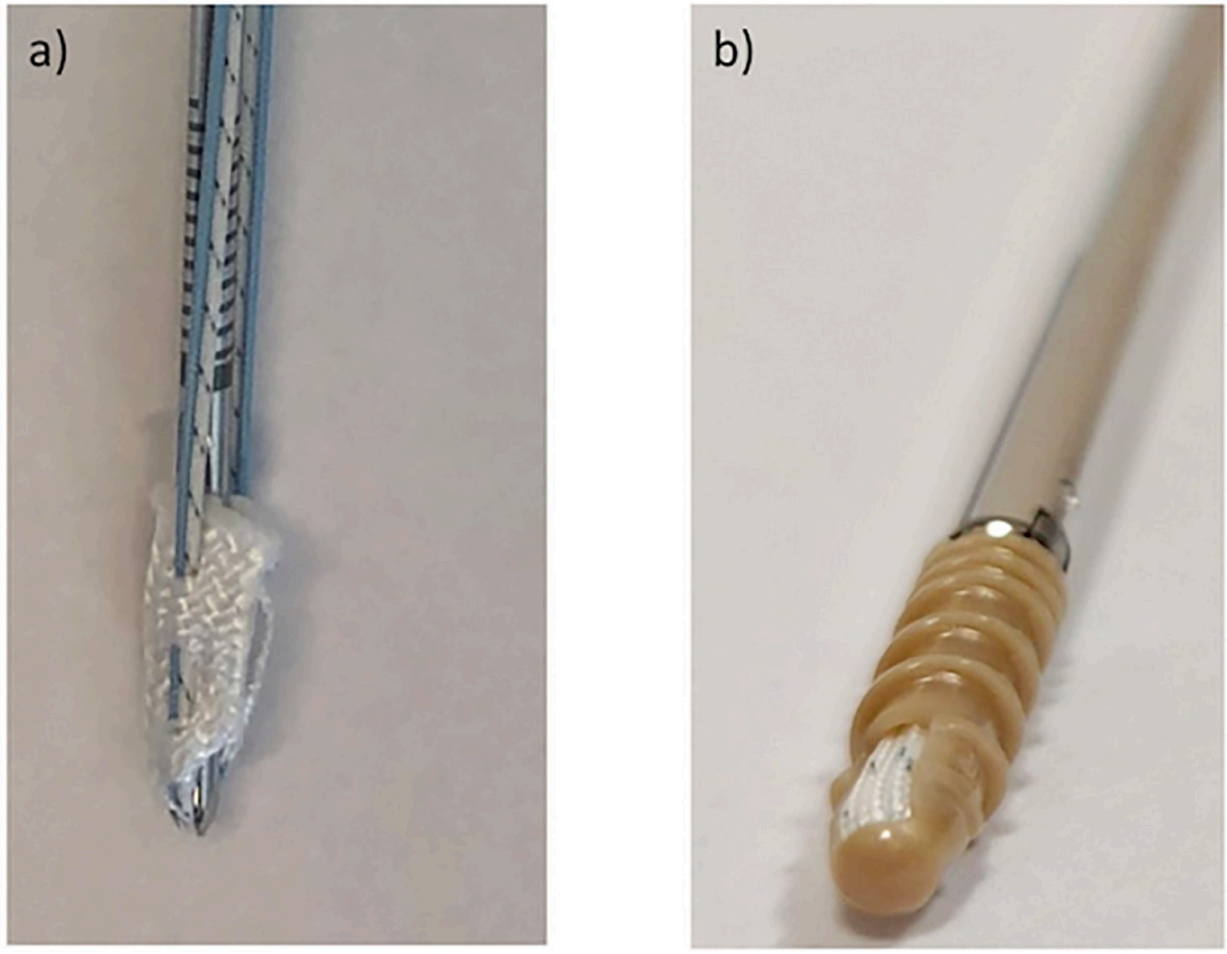
Presentation of the two anchor types used in this study: a) all-suture anchor b) conventional anchor.

The all-suture anchor is entirely composed of suture material containing ultra-high-molecular-weight polyethylene and designed as a tape through which the suture is woven (Fig 1). Through direct pull of the surgeon the anchor deploys to 5 mm and is fixed underneath the smaller pilot hole and cortex [8]. The conventional anchor is made of the non-degradable plastic polyetheretherketone (PEEK) and provides a fully threaded screw type design (Fig 1).

This PEEK anchor was chosen because it was shown to provide a strong fixation and offers a second interleaved thread placed at the proximal end to maximize cortical compression [22]. This is of particular importance since a missing intracortical fixation could result in different moment arms which may lead to the previous described toggling. Moreover it has been associated with suture cutting through the bone when subjected to different insertion angles [18,23]. Furthermore, non-degradable PEEK anchors have become a commonly utilised material choice since in contrast to metal anchors PEEK anchors do not interfere with imaging and allow for simplified revision as it can be drilled through [24].

Anchor implantation was performed according to the manufacturers’ instructions within the proximal anterior part of the greater tubercle, 1 cm posterior of the bicipital groove as previously described [5,7,25]. For the Y-Knot RC^®^ the second of the different circumferential laser-marks on the inserter was chosen assuring a similar anchor depth in each sample. Each of the three groups was assigned to a specific anchor implantation angle of either 45°, 90° or 110°. These values were chosen since they represent the range from 45° to the most obtuse angle. The previously published angle of 135°, was not attainable due to the anatomical impediment of the acromion [16]. Prior to testing the specimens were defrosted. The humerus was perpendicularly transected at the mid-diaphyseal level and all overlaying soft-tissue was dissected to expose the bone surface of the greater tubercle, the junction of the greater tubercle and the humeral head articular surface.

**Fig 2:**
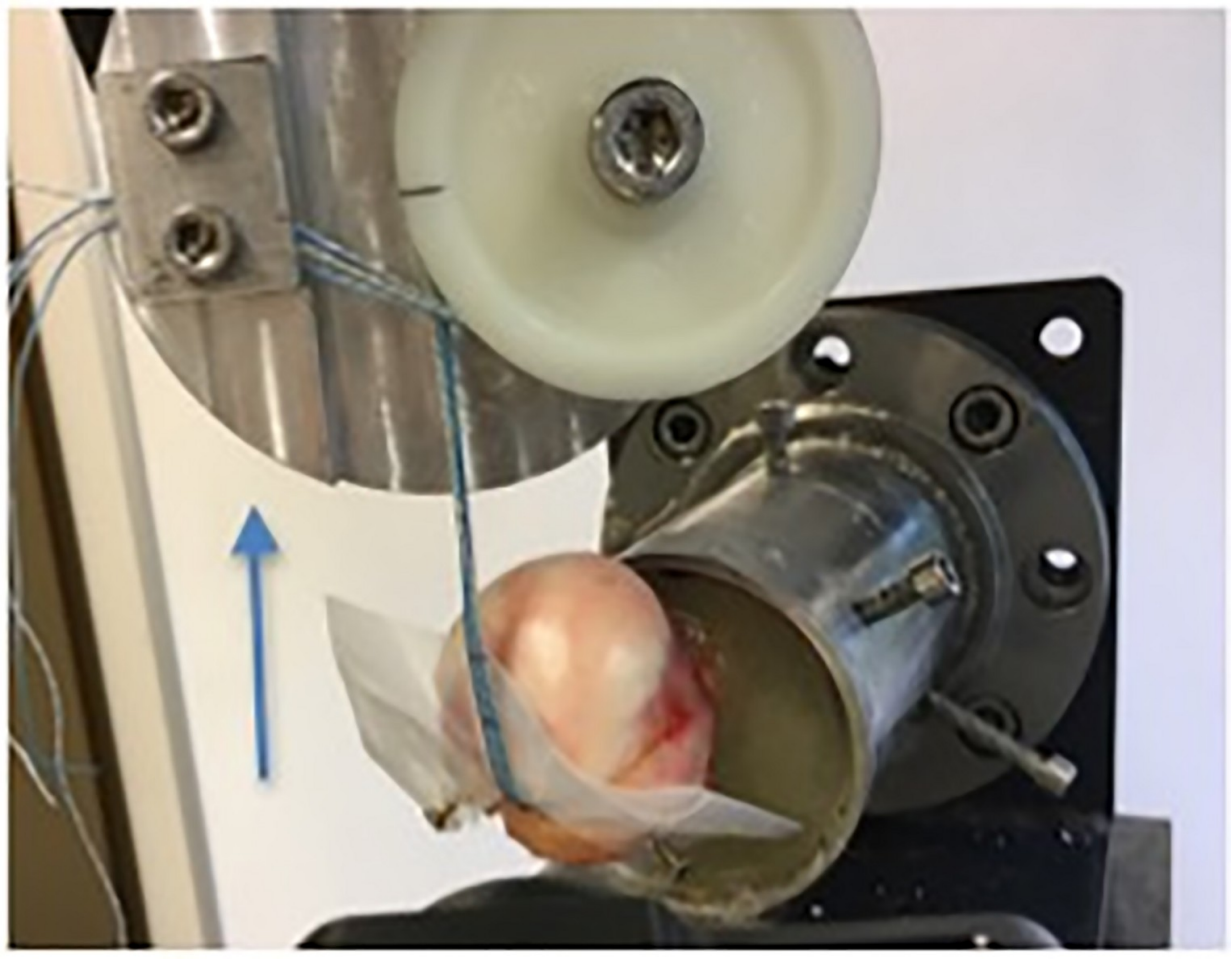
Experimental set up. The blue arrow represents the direction of load application.

The distal end of the specimen was potted upright in a steel tube and fixed using a Methylmethacrylat solution (Technovit 4004, Hereaus Kulzer, Hanau, Germany). Fixation was performed up to 2cm proximal of the lower humeral bone-cartilage junction. Throughout, the specimens were wrapped in moist tissue in order to preserve the constitution of the tissue. The experiments were performed at room temperature. After anchor insertion at the predefined angle (45°-90°-110°) the emerging suture threads were wrapped around a pulley and secured with clamps. To ensure an exact angle of insertion in each sample, two platforms with a defined angle of 45 and 110° were constructed. The humeral heads were then perpendicularly fixed to the chosen platform so that every anchor could be inserted in the same predefined angle. A clamp to anchor distance of 10 cm was chosen as previously published [7]. Since the load was applied by the threads and not the cuff muscle, the humeral head was protected by a plastic cover. This avoided the previously reported unphysiological suture failure by cutting through the bone [26]. Biomechanical testing was performed using a servo-hydraulic testing machine (MTS 858.2, MTS Systems, Eden Praire, Minnesota; Fig 2).

Loading protocol was designed to simulate rehabilitation after rotator cuff repair as follows: After an initial preload with 20N a cyclic testing protocol with a step-wise increasing pullout of 0.05N for each cycle at a rate of 1Hz was performed. Cyclic extension was continued until system failure. The number of completed cycles, the maximum pullout force, failure mode, anchor displacement and construct stiffness were recorded. Displacement was defined as difference between the initial construct length after pre-load and the subsequent clamp to anchor distance at 1000, 2000 and 3000 cycles. Two samples (2/30)–one with an all-suture and one with a conventional anchor–had to be excluded in the analysis since technical failure occurred rendering the specimen unusable for the experiments. Video analysis ensured a reliable follow through.

Ethical Review Committee Hamburg, Germany; study number: WF-27/17.

Statistical analysis: ANOVA analysis were performed using SPSS (Version 21, IBM, Armonk, USA) with a significance level of α = 0.05. In addition, the measured forces for the left and right humerus of the same donor were used in a non-parametric paired Wilcoxon-Test to exhibit potential advantages of one suture method independent of the implantation angle.

## 3. Results

The usage of matched pairs caused comparable vBMD for both treatments (all-suture anchor vs. conventional treatment) in all three groups (n = 10 specimens per implantation angle, Table 1).

**Table 1:** Received BMD in the different groups partitioned according to the anchor implantation angle and the anchor type.

	vBMD total [mg/cm^3^]	vBMD all-suture [mg/cm^3^]	vBMD conventional [mg/cm^3^]
45° Implantation	126 ±18 n = 10	124 ±16 n = 5	128 ±22 n = 5
90° Implantation	126 ±26 n = 10	127 ±30 n = 5	126 ±25 n = 5
110° Implantation	127 ±16 n = 10	127 ±14 n = 5	128 ±19 n = 5

Two different types of failure modes were observed. All of the systems, except for one sample failed by anchor pullout (Table 2). Suture Breakage was only seen in one sample using the conventional anchor system with an implantation angle of 45° (Table 2).

**Table 2:** Final pullout strength, number of completed cycles at system failure and absolute numbers of anchor pullout as failure mechanism.

	Final pullout strength (N)	Completed number of cycles at pullout	Anchor pullout
Mean conventional anchor (n = 14)	259 ± 61	4093 ± 1142	13/14
Mean all-suture anchor (n = 14)	247 ± 78	3791 ± 1349	14/14
p-value	0.65	0.52	

Comparing the conventional and the all-suture anchors regardless of the angle of implantation reveals no significant difference in maximum pullout strength or in the number of completed cycles (Tables 2 and 3). Furthermore, Wilcoxon-Test analysis regarding potential advantage of one suture method in terms of pullout strength independent of the implantation angle did not show any significant differences in between all-suture and conventional anchors (p = 0.460).

**Table 3:** Absolute numbers of completed cycles for each anchor type at 1000, 2000, and 3000 cycles.

	1000 cycles	2000 cycles	3000 cycles
All-suture anchor (n = 14)	14	14	10
Conventional anchor (n = 14)	14	13	13

**Fig 3:**
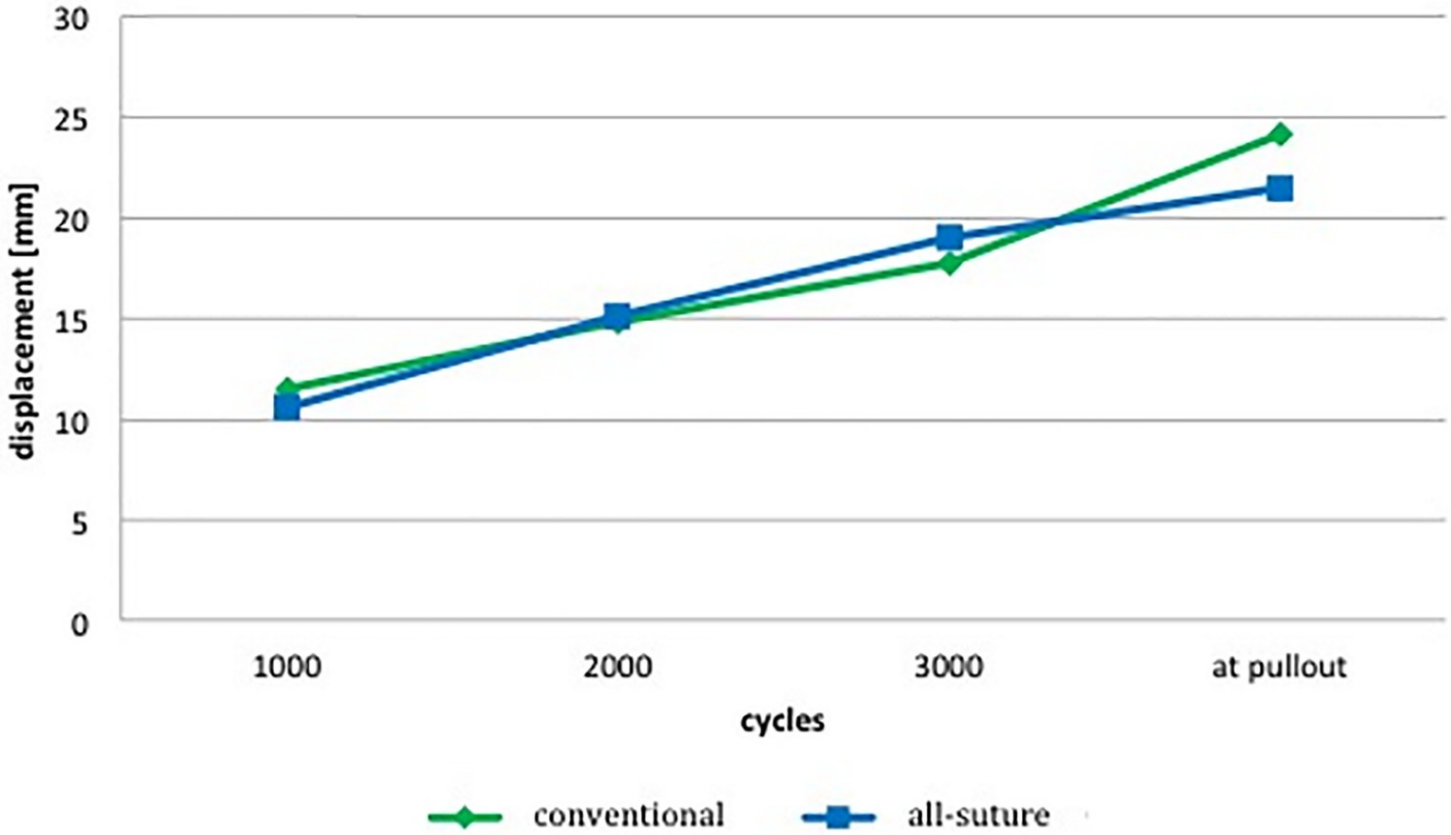
Displacement at 1000, 2000, 3000 completed cycles and right before pullout. No significant difference in between the all-suture and the conventional anchor system could be seen. See Table 3 for their numerical value.

No significant changes could be detected comparing the conventional and the all-suture anchor system (Fig 3) regarding displacement. Obtained Data for >3000 cycles were not included in the stiffness and displacement analysis since most anchors had already been pulled out. Thus yielding sufficient data plots for further analysis was not possible.

**Fig 4:**
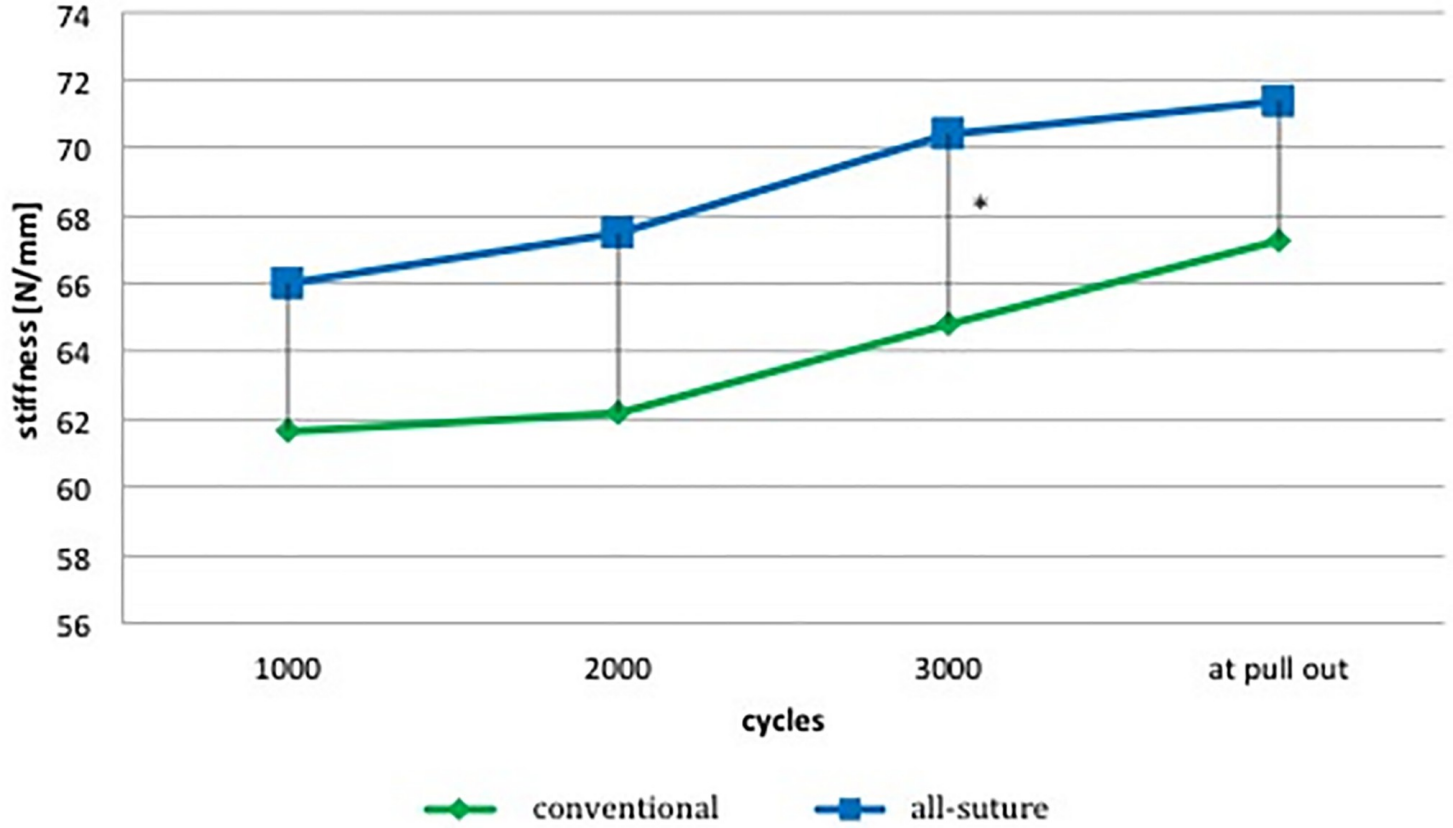
Mean stiffness at 1000, 2000, 3000 completed cycles and right before pullout. * indicates statistical significance. Numerical values see Table 3.

All-suture anchor systems show an increased stiffness compared to the conventional anchors. Statistical significance though could only be detected at 3000 cycles (Fig 4).

**Fig 5:**
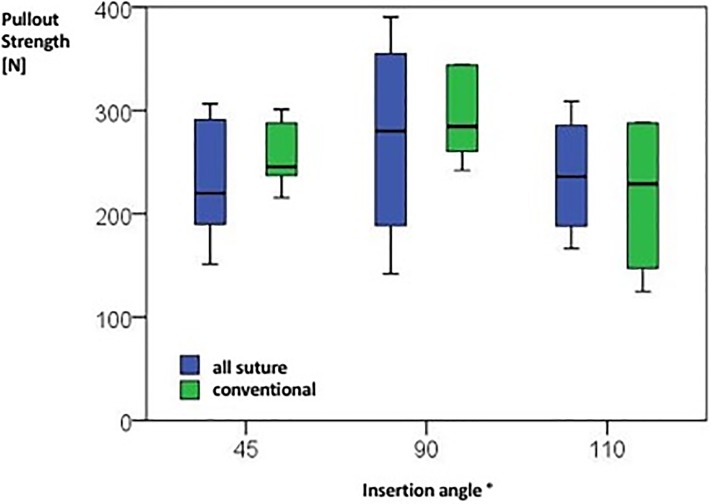
Boxplot analysis showing maximal cyclic pullout strength in Newton for each anchor type depending on anchor insertion angle. Statistical significance could neither be observed for the different insertion angles nor for the anchor type. Numerical values see Table 1.

Maximum force could be detected at a 90° angle in both anchor types (Fig 5, Table 1). Inserting them in either more acute (45°) or obtuse (110°) angles reduces pullout strength regardless of the type. However, those differences are not statistically significant. Analyzing data for displacement, stiffness and completed cycles depending on the implantation angle as well as anchor type reveals no statistical significance in either category (Table 4).

**Table 4:** Mean completed cycles at pull out as well as stiffness and distance at 1000 cycles for all three implantation angles and both anchor types. 1000 completed cycles was chosen, since all samples could be included. No statistical significance was detected in either category.

	Completed cycles at pull out	Stiffness at 1000 cycles [N/mm]	Distance at 1000 cycles [mm]
45° Implantation			
• Conventional anchors (n = 5)	4142 ± 480	59.8 ±5.6	10.1 ±1.9
• All-suture anchors (n = 5)	3558 ± 1143	65.9 ±15.2	9.3 ±2.3
90° Implantation			
• Conventional anchors (n = 5)	4811 ±807	60.2 ±6.6	11.8 ±1.9
• All-suture anchors (n = 5)	4271 ± 1848	65.0 ±8.4	11.1 ±2.1
110° Implantation			
• Conventional anchors (n = 4)	3133 ± 1543	65.8 ±6.5	12.9 ±3.3
• All-suture anchors (n = 4)	3484 ± 995	67.4 ±2.8	11.5 ±2.4

## 4. Discussion

This study compared the biomechanical properties of two different anchor systems in a cyclic loaded human humeral head ex vivo model. There was no significant difference between the conventional and the all-suture anchor system in neither of the recorded results. These findings are consistent with previously published papers showing comparable biomechanical properties of the two anchor types in a quasi-static setup [11–14,27].

Goshka et al. tested an all-suture system in a human double row rotator cuff repair model and Galland et al. another all-suture system in a bovine humerus model. Both showed similar results compared to solid body anchors, supporting the findings of this study. Comparing the properties of the all-suture anchors used in these two studies to the present results, the maximum pullout force for the double row repair was 313N compared to 265N reported by Galland et al. and 259N in this set-up. Overall, the findings show comparable results for the three different all-suture anchors with the present all-suture system ascertaining the lowest maximum pullout force. Considering that pullout strength depends on the number of inserted anchors the slightly higher values yielded in the other studies could be well explained, since experiments were performed using two anchors in the study by Galland et al. and four anchors in the study by Goshka et al. [11,12,28]. So, while these comparable findings strengthen the results, comparison is limited due to additional tendon-anchor interface, different bone types and usage of multiple anchors.

Galland et al. used uniaxial pullout tests and Goshka et al. limited the cyclic pull out force to 500 cycles, followed by linear pull out. The present test set-up mimics a more realistic scenario instead, since a continues cyclic loading was used which reflects characteristic rehabilitation and long term stress [11,12]. Continuous cyclic loading could be another reason for the slightly lower pull out force of the all-suture anchor in this study. This is emphasized by the fact that the conventional anchor yielded lower values as well, compared to previous studies [22]. Still, the endurance shown by both anchor types in this study could be crucial when it comes to rehabilitation and long-term stability. Previously reported numbers for pull out strength might not suitably exhibit the absolute pull out force since neither anatomically nor biologically accurate models were used [7–9,13]. Therefore, all-suture anchors could withstand the previously estimated maximum force of 250N and higher that must be withstood in vivo. Especially considering that due to the smaller size, an increased number of anchors can be placed in the ROI ensuring placement safety at even higher pull out forces [9,11,12,29–31].

In contrast, Nagra et al. recently questioned the benefit of all-suture anchors. They demonstrated a decreased failure load and increased total displacement for all-suture anchors compared with conventional anchors even though a similar human humeral cadaveric model was used. No sufficient selection regarding the bone quality was performed though, which could lead to bias. In the present study, this was minimized by determining the vBMD of all samples and forming appropriate groups. Moreover, neither the direction of pull nor the angle of implantation was properly defined, contrary to this study. Differing results could also be explained by the discrepancy between the varying all-suture anchor systems. Even though the basic principle includes an expansion of the anchor and its locking underneath the cortex, significant differences have recently been reported [7–9,32]. Still, comparing the pullout strength of similar all-suture systems, the results of this study were superior to the ones reported by Nagra et al. (247.2 ± 78 N vs. 145.8 ± 23.1 N). Analyzing the protocols to further evaluate the discrepancy revealed a cycle limitation of 200 and different pretensions (10N in the study of Nagra et al. vs. 20N in the present study). This could be another explanation for the difference since Dwyer et al. previously showed that an increased pretension strengthens the all-suture pull out force [7,13].

Focusing on possible anchor displacement Nagra et. al as well as Pfeiffer et al. announce higher displacement for all-suture anchors compared to conventional anchors [7,27].

Furthermore, in a biomechanical study by Mazzoca et al. conventional anchors required significantly higher loads to achieve 2 mm of displacement compared to all-suture anchors [14]. In all three studies, just a single all-suture system showed significant higher displacement though whereas all other all-suture systems which were tested did not deviate significantly from the conventional control group. Again, different properties among all-suture systems themselves could be a crucial point.

Displacement at pull out in the present study (mean 21.5 mm) is in accordance to Nagra’s results (mean 23.6 mm). The present results demonstrate equivalent anchor displacement for the all-suture and the conventional anchors which therefore questions the hypothesis that all-suture anchors suffer greater displacement, especially considering that this protocol provides higher cycle rates than previous studies [7,14,27]. Direct translation of the obtained data however is not possible since recorded measurements represent displacement of the whole system including the suture material and not just isolated anchor failure.

The fact that all-suture anchors have similar biomechanical qualities to conventional anchors is underlined by the stiffness analysis. All-suture anchors show not just equal but even increased stiffness compared to the conventional anchors at each recorded cycle (p<0.05 at 3000 cycles).

One main aim of this study was to further evaluate the optimum angle of insertion in both anchor systems. All published data focuses on conventional anchor systems so far. Burkhart et al. first defined the optimum insertion angle as 45° or less based on mathematical principles in 1995 [15]. Following these findings, several authors found opposing results in a variety of different biomechanical set ups [3,5,19,33]. Clevenger et al. and Strauss et al. suggested a windshield wiper type motion as failure mode for anchors placed at more acute angles, therefore challenging Burkhart’s theory [3,5]. Additionally, Green et al. combined different insertion angles with different pull out angles in polyurethane foam blocks and claim that the optimum insertion angle should be parallel to the applied load [19]. Still, comparison of these studies remains difficult, since some describe the angle of the anchor relative to the bone while others examine the angle of the force vector (pull of the rotator cuff) with respect to the bone [34]. Moreover, the lack of intracortical anchor fixation questions these results since different moment arms could lead to the described toggling [16,18].

In this study, fully threaded anchors with intracortical fixation at three different insertion angles relative to the bone surface were therefore tested. Additionally, a physiological 90° direction of pull with respect to the bone assuring similar settings in each experiment was used. The results show that an implantation angle of 90° resists the highest pull out forces, while no statistical significance could be reached. Still, the present data contradicts the theory of favoring an implantation angle of 45° [16]. Even though the mathematical explanation is right in theory it cannot be translated to an in vivo clinical scenario. In fact, the present study supports the opinion that the insertion angle of conventional anchor systems may clinically not be as important as it is thought to be.

As hypothesized, a significant difference between different implantation angles could not be observed, which we attribute to the morphology and functional properties of ASA. Obtained data suggests that the implantation angle of all-suture anchors does not influence pull out characteristics. Still an angle of 90° reached the highest pullout strength which could be explained by a symmetric fixation underneath the cortex which reflects the principle of all-suture anchors [7].

Limitations of this study include the lack of in vivo conditions as well as the missing suture tendon interface as another possible failure mechanism. Since only two different anchors were used inter-anchor variability is not respected. Still this limitation is due to variations in product design and therefore hardly to exclude. The present protocol includes a more realistic unlimited cyclic loading. Therefore, comparison with other studies using linear pullout tests is incoherent.

Furthermore, the limited number of human specimen due to availability and its influence on the power of the study needs to be noticed. In the present study, the exhibited differences between conventional anchors and all-suture anchors were rather small (2–15% of the mean value), especially if compared to the standard deviation within each group (7–38% of the mean value) and no statistical significant differences could be exhibited. Consequently, the powers of the experiments were small. Taking the mean observed effect size of 10% and the relative standard deviation of about 20%, the power analysis (α = 0.0.5 / β = 0.2) requested a sample size of 50 specimens per group to prove if accepting the null hypotheses–differences are negligible—were appropriate or not. However, we believe that only large differences (that can be spotted with those kinds of experiments) are relevant for the multifactorial situation that occur in vivo.

Finally, a static humerus fixation was used which does not reflect the dynamic change of forces the anchor experiences in vivo. Therefore, clinical transferal of the results should be performed carefully.

In conclusion, this study reveals that all-suture anchors show similar biomechanical properties compared to conventional anchors and therefore could improve clinical work especially due to decreased bone damage. Still, lack of knowledge regarding the impact of the all-suture anchor on the humeral bone and its surrounding, once it has been placed, indicates the need for further human morphological and histological studies.

Based on the present findings 90° seems to be the implantation angle with the best biomechanical qualities for all-suture anchors as well as for conventional anchors. Still lack of significance indicates that a specific implantation angle does not seem to be as relevant as previously reported.
